# Enzymes for *N*-Glycan Branching and Their Genetic and Nongenetic Regulation in Cancer

**DOI:** 10.3390/biom6020025

**Published:** 2016-04-28

**Authors:** Yasuhiko Kizuka, Naoyuki Taniguchi

**Affiliations:** Disease Glycomics Team, Systems Glycobiology Research Group, Global Research Cluster, RIKEN, Wako, 351-0198, Japan; y.kizuka@riken.jp

**Keywords:** cancer, fucose, GlcNAc, *N*-Glycan, glycosylation, glycosyltransferase

## Abstract

*N*-glycan, a fundamental and versatile protein modification in mammals, plays critical roles in various physiological and pathological events including cancer progression. The formation of *N*-glycan branches catalyzed by specific *N*-acetylglucosaminyltransferases [GnT-III, GnT-IVs, GnT-V, GnT-IX (Vb)] and a fucosyltransferase, Fut8, provides functionally diverse *N*-glycosylated proteins. Aberrations of these branches are often found in cancer cells and are profoundly involved in cancer growth, invasion and metastasis. In this review, we focus on the GlcNAc and fucose branches of *N*-glycans and describe how their expression is dysregulated in cancer by genetic and nongenetic mechanisms including epigenetics and nucleotide sugar metabolisms. We also survey the roles that these *N*-glycans play in cancer progression and therapeutics. Finally, we discuss possible applications of our knowledge on basic glycobiology to the development of medicine and biomarkers for cancer therapy.

## 1. Introduction

Protein glycosylation, the most abundant post-translational modification, is deeply involved in cellular and organ homeostasis [1]. The cellular glycosylation machinery is often affected by the development of diseases including cancer, leading to the emergence of disease-specific changes in the glycome [2,3,4]. These glycan changes are not only functionally involved in disease processes, but can also be exploited to distinguish specific diseases as biomarkers. However, regarding cancer biomarkers and therapy, only a few glycan-targeted or glycoform-specific markers are currently in clinical use [2]. To clinically apply basic knowledge on glycobiology to cancer therapy, there is a need for a deeper understanding of the roles of glycans in cancer and their therapeutic potential.

*N*-glycan, the main subject of this review is one of the two major classes of protein glycosylation (the other is *O*-glycan) and occurs at the Asn-X-Ser/Thr sequon (X is any amino acid except Pro) [5]. In mammals, after the first *en bloc* transfer of *N*-glycan composed of 14 saccharides to a nascent protein in endoplasmic reticulum (ER) [5], it is processed into a mature form by undergoing several trimming and transfer reactions by glycosidases and glycosyltransferases in the Golgi apparatus. To date, ~200 glycosyltransferase genes have been cloned, and by cooperating and sometimes competing with each other, the enzymes coded by these genes produce complex *N*-glycan structures on target glycoproteins in cells.

**Figure 1:**
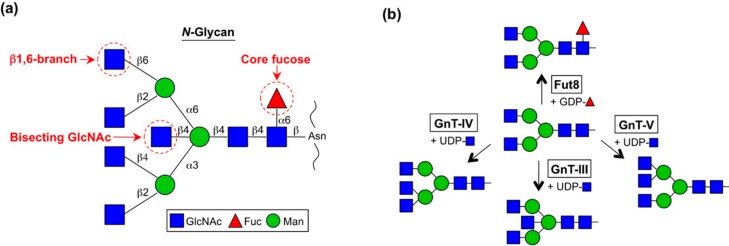
Schematic diagram of *N*-glycan branches and enzymes responsible for their synthesis. (**a**) Structure of *N*-glycan branches; (**b**) Specific enzymes responsible for the biosynthesis of *N*-glycan branches.

One of the unique features of *N*-glycan structures is the large number of potential branches. All mammalian *N*-glycans have the common core pentasaccharide [Manα1-6 (Manα1-3) Manβ1-4GlcNAcβ1-4GlcNAc-Asn] and multiple branches are formed on this core structure by the actions of several GlcNAc transferases (GnTs) and a fucose transferase, Fut8 (Figure 1) [6]. As in other types of glycosylation, *N*-glycan structures including branches are often altered in cancer cells by several mechanisms.

In this review, we particularly focus on *N*-glycan branches. We first describe the cancer-related changes in the expression of glycosyltransferases responsible for the synthesis of *N*-glycan branches and their functional outcomes. Next, we focus on the mechanisms by which these branches are aberrantly synthesized in cancer by genetic, epigenetic and metabolic mechanisms. Finally, we describe potential clinical applications of these *N*-glycan branches to cancer therapy and diagnosis.

## 2. Enzymes for Branch Synthesis and Their Involvement in Cancer

### 2.1. GnT-III (MGAT3)

**Figure 2:**
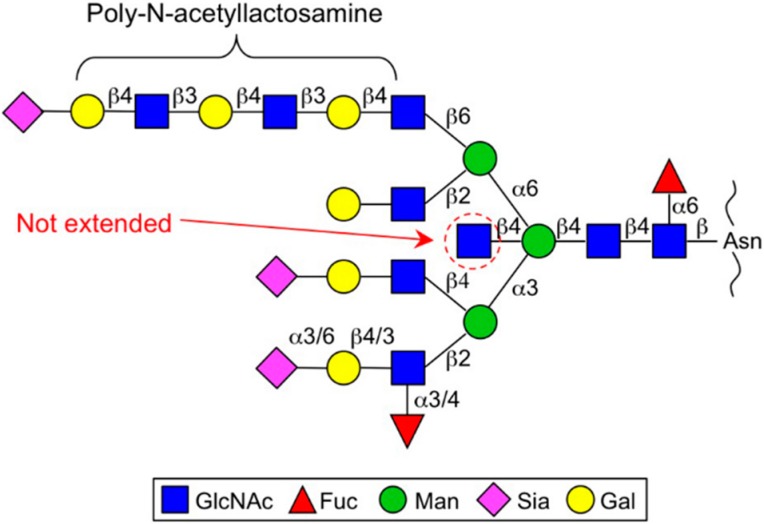
Typical multi-branched *N*-glycan structure. Bisecting GlcNAc (indicated by a red dashed circle) is not further elongated, whereas other GlcNAc branches are usually elongated by Gal and Sia. The β1,6-branch sometimes carries repeating *N*-acetyllactosamine units (polylactosamine).

GnT-III catalyzes the transfer of a GlcNAc residue to the β-mannose via the β1,4-linkage to make a so-called “bisecting GlcNAc” structure (Figure 1) [7]. Bisecting GlcNAc has unique features that differ from those of other GlcNAc branches [8]. First, bisecting GlcNAc is not or barely elongated, whereas other GlcNAc branches in *N*-glycan are further decorated with Gal, Sia, Fuc and others (Figure 2). The second feature of bisecting GlcNAc is its negative impact on the activities of other glycosyltransferases. The enzymes responsible for producing other *N*-glycan branches (e.g., GnT-IV and GnT-V) are often inhibited almost completely by the presence of a bisecting GlcNAc residue in *N*-glycan [5,9,10]. This unique feature gives rise to a complex situation regarding glycan structure because the upregulation of GnT-III could affect the formation of other *N*-glycan structures as well as an increase in bisecting GlcNAc. Therefore we have to bear in mind that up and downregulation of GnT-III protein could change the overall *N*-glycan structures of a protein or cell of interest. Recently it was suggested by molecular dynamics simulation and crystallographic studies that the presence of bisecting GlcNAc has a major impact on the conformer selection of glycans and restricts *N*-glycan conformation to a back-fold type as a preferable conformer [11,12,13,14]. This effect may explain the molecular basis for several other glycosyltransferases being unable to act on bisected glycans.

The activity of GnT-III was first identified in the early 1980s [15,16]. Subsequently, our group biochemically purified this enzyme from rat kidney and identified its gene and cDNA [17]. It is noteworthy that normal liver and hepatocytes do not or barely express GnT-III, while drug-induced liver cancer tissue and hepatoma cells express it at high levels [18]. In addition, GnT-III (*MGAT3*) transcripts or its product bisecting GlcNAc are aberrantly upregulated in some other cancer cells including ovarian cancer [19] and leukemia [20], suggesting that bisecting GlcNAc plays certain roles in cancer development and progression.

To examine the role of bisecting GlcNAc in cancer *in vivo*, we injected B16 melanoma cells overexpressing GnT-III to syngeneic nude mice and analyzed their metastatic potential. We found that the GnT-III transfectant showed significantly less metastasis in mouse lung, suggesting that the overproduction of bisecting GlcNAc can suppress cancer metastasis [21]. Furthermore, Pamela Stanley’s group revealed that GnT-III (*Mgat3*)-deficient mice showed increased tumor growth and metastasis in a polyoma middle T (PyMT)-induced breast cancer model [22]. These results suggest that bisecting GlcNAc reduces cancer growth and aggressiveness. Several mechanisms underlying these phenomena have been suggested. First, the overexpression of GnT-III results in the prolonged cell surface retention of E-cadherin, leading to increased cell adhesion [23]. E-cadherin itself was also reported to upregulate the level of GnT-III, thereby forming a positive feedback loop between E-cadherin and GnT-III that promotes cell adhesion [24,25]. Several cell adhesion molecules are also functionally modified with bisecting GlcNAc including cadherins, laminins and integrins [26,27,28,29]. In addition, the expression level of bisecting GlcNAc is downregulated during epithelial-messenchymal transition (EMT) and this downregulation is critical for the process of EMT [30,31]. These lines of evidence show that bisecting GlcNAc regulates cancer metastasis by controlling cellular adhesion. Second, the overexpression or knockout of GnT-III might affect the overall synthesis of *N*-glycan as suggested by the enzymatic properties of other GnTs. In particular, as the GnT-V-producing β1,6-branch on *N*-glycan promotes cell proliferation and growth, as described below, bisecting GlcNAc may indirectly regulate cancer cell properties by inhibiting other glycosyltransferases including GnT-V. Furthermore, Jianguo Gu’s group recently reported that GnT-III overexpression also inhibited α2,3-sialylation [32], although the mechanisms behind this downregulation of α2,3-Sia remain unclear. The suppressive effect of bisecting GlcNAc on tumor metastasis was also shown to be highly dependent on the cellular sialylation status [32], indicating the importance of obtaining an overview of *N*-glycan structures when we examine the properties of cancer cells.

Surprisingly, GnT-III-deficient mice were found to show retarded tumor progression in a diethylnitrosamine-induced liver cancer model, probably due to reduced hepatocyte proliferation in the mutant mice [33]. Moreover, GnT-III-overexpressing K562 leukemia cells colonized the spleen in nude mice more than control cells did, owing to enhanced evasion from NK cell immunity [34]. These results suggest that GnT-III does not always act as a tumor-suppressing factor, but sometimes works to enhance cancer progression depending on the cancer type and environment around a tumor. Although the reasons for this discrepancy is unclear at present, it suggests that bisecting GlcNAc has a wide range of functions and that different cellular phenotypes may arise from distinct target proteins expressed in each cell type.

We recently found that GnT-III-deficient mice exhibited reduced amyloid-β generation in the brain and a great improvement of Alzheimer’s disease pathology, with improved performance in short-term memory tasks [35,36]. These findings seemed to be caused by the abnormal lysosomal localization of a key protease, β-secretase (BACE1) in the mutant mice, in contrast to its localization in the early endosomes in wild-type control. Although the precise mechanism by which the endosomal localization of BACE1 is regulated by bisecting GlcNAc is still unclear, this suggests that bisecting GlcNAc modification has the potential to control the intracellular trafficking of target proteins. Large-scale identification of bisecting GlcNAc-containing glycoproteins and their characterization in terms of cellular localization might reveal how bisecting GlcNAc serves as a trafficking tag for its target proteins, which could also be involved in cancer-related cellular events.

### 2.2. GnT-V and GnT-IX (GnT-Vb) (MGAT5 and MGAT5B)

GnT-V catalyzes the transfer of a GlcNAc residue to the α1,6-mannose via the β1,6-linkage to make the so-called “β1,6-GlcNAc branch” structure (Figure 1) [37]. This enzyme was first purified by Michael Pierce’s group from rat kidney [38,39] and subsequently by our group from a human lung cancer cell line [10]. Many reports have revealed that the levels of *MGAT5* mRNA and its enzymatic product β1,6-branch are aberrantly upregulated in cancer cells and that this β1,6-branch is highly involved in cancer growth and metastasis. The transcriptional mechanisms of oncogenic upregulation (defined hereafter as upregulation by oncogene or its downstream factor) of the GnT-V gene are described below (see Section 3.1 “Genetics”).

β1,6-GlcNAc is normally further modified with a β1,4-Gal residue to make an *N*-acetyllactosamine (LacNAc) structure and this LacNAc unit is sometimes repeatedly synthesized by the sequential action of β3GnT and β4GalT to make a polylactosamine structure, which is considered to act as a high-affinity ligand for galectins (Figure 2) [40]. Galectin-glycan interaction has been proposed to hold the target glycoproteins, such as growth factor receptors at the cell surface and prolong their localization there before endocytosis, which in turn augments growth factor signaling and cell proliferation [41]. Based on this concept, GnT-V is assumed to regulate cell proliferation by controlling the surface retention time of the target proteins. Moreover, the β1,6-branch also regulates the function of other cancer-related molecules such as matriptase, tissue inhibitor of metalloproteinase-1 (TIMP-1) and E-cadherin. Matriptase, a serine protease involved in cancer invasion and metastasis, was found to be stabilized and constitutively activated by β1,6-branch modification, probably contributing to the enhanced invasiveness of gastric cancer cells [42,43]. Specific *N*-glycosylation sites of E- and *N*-cadherin are also the target sites for GnT-V action, leading to the increased cell migratory/invasive phenotypes of various cancer cells [44,45]. As described above, GnT-V action is inhibited by the presence of bisecting GlcNAc. Therefore, regulation of the functions of these target proteins by GnT-V might compete with that by GnT-III in cancer [46]. Intriguingly, GnT-V itself has a unique basic motif like vascular endothelial growth factor (VEGF) and plays a role in angiogenesis [47]; this function was shown to occur irrespective of its catalytic activity. It is well known that glycosyltransferases including GnT-V are secreted and present in body fluids in a soluble form [48,49] and recent reports have demonstrated that Signal peptide peptidase-like 3 (SPPL3) is a major protease responsible for GnT-V secretion (as described below) [50,51]. Considering the fact that angiogenesis is a critical step for cancer progression, the nonenzymatic angiogenic function of GnT-V could also promote tumor progression *in vivo*.

GnT-V (*Mgat5*)-null mice appeared normal but exhibited reduced tumor growth and progression in a PyMT-induced mammary tumor model [52], showing that the β1,6-branch plays key roles in tumor progression *in vivo*. GnT-V-null mice also showed a delay of tumor onset and a reduction of tumor-initiating cells in HER-2/neu-induced mammary tumors [53]. Conversely, the overexpression of GnT-V in normal mammary epithelial cells (MCF-10A) resulted in an increase in cell proliferation and abnormal morphogenesis in 3D culture, an early characteristic of mammary neoplastic transformation [53]. Furthermore, *MGAT5* transcripts and/or their products, β1,6-branch, are often upregulated in many types of cancer, such as gastric [54], esophageal [55], colon [56] and liver cancers [57]. The increased expression of β1,6-branch was also shown to be correlated with the poor prognosis of breast cancer [58]. This accumulating evidence strongly supports the notion that GnT-V plays pivotal roles in cancer onset, growth and metastasis, and is a promising drug target for cancer therapy.

**Figure 3:**
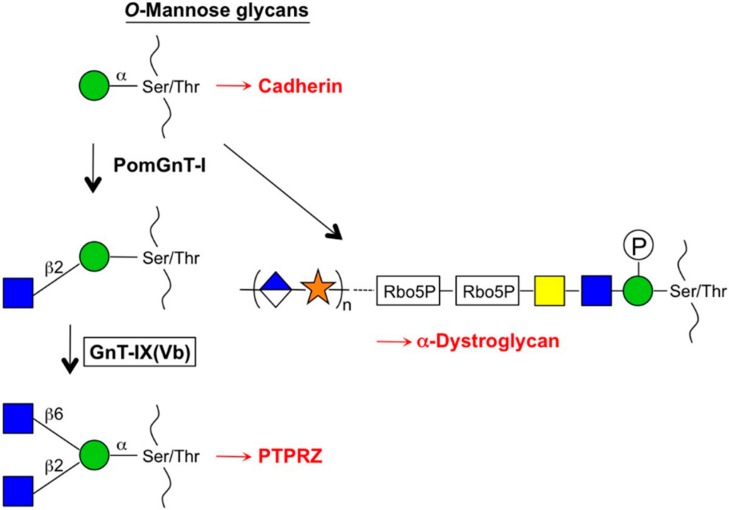
Branch formation in *O*-mannose glycans. GnT-IX acts on an *O*-mannose glycan after the action of PomGnT-I. Branched *O*-mannose glycan is expressed on a phosphatase, PTPRZ, and regulates remyelination in the brain. Phosphorylated and elongated *O*-mannose glycan is attached to α-dystroglycan, which is essential for muscle functions.

In humans, a cDNA homologous to *MGAT5* that encodes the enzyme GnT-IX(Vb) named *MGAT5B*, was cloned independently by Michael Pierce’s group and our group [59,60]. The GnT-IX enzyme shows the same *N*-glycan branching activity as GnT-V but the K_m_ value for *N*-glycan substrate is quite high [61]. Instead, this enzyme showed efficient transfer activity toward *O*-mannose glycan with high affinity [62] suggesting that GnT-IX has intrinsic functions that differ from those of GnT-V and acts as a branching enzyme for *O*-mannose glycans (Figure 3) [63]. This assumption was supported by the unique and exclusive expression of GnT-IX mRNA in the brain [64,65]. In addition, mass-spectrometry (MS) analyses of *N*-glycans and *O*-mannose glycans from GnT-V (*Mgat5*)- and GnT-IX (*Mgat5b*)-null mouse brains actually revealed that GnT-V and GnT-IX predominantly synthesize β1,6-branch in *N*- and *O*-mannose glycans respectively, although it remains a possibility that GnT-V weakly acts on *O*-mannose glycans. Interestingly, GnT-IX-null mice exhibited improved remyelination in the brain after cuprizone-induced demyelination [66]. It has been proposed that this phenotype is caused by the attenuation of reactive astrocytes in the mutant mice after injury, and a potential target glycoprotein for this phenomenon, which was modified by GnT-IX, is receptor-type protein phosphatase-zeta (PTPRZ). Increased dimerization and reduced phosphatase activity of PTPRZ were also observed after GnT-IX overexpression in neuroblastoma cells, which resulted in decreased cell adhesion and increased migration [67], suggesting that GnT-IX plays some roles in the regulation of cell adhesion. Notably, *O*-mannose glycans were also shown to be attached to cadherins and to regulate cell adhesive property [68,69]. In addition, because defects in the synthesis of *O*-mannose glycans cause severe genetic muscular disorders (referred to as α-dystroglycanopathies) [70,71], the biosynthetic pathway for *O*-mannose glycans has received a lot of attention and is an actively studied topic in the field of glycobiology (Figure 3). Regarding cancer, *MGAT5B* was reported to be highly expressed in neuroblastoma cells [72]. It was also reported that *MGAT5B* was identified as a highly upregulated gene in human prostate cancer cells upon metastasis in a mouse xenograft model [73]. In addition, *MGAT5B* seems to be aberrantly upregulated in various non-brain tumors by epigenetic mechanisms [74]. These results suggest that GnT-IX is involved in cancer progression but its detailed roles in cancer still remain to be elucidated.

### 2.3. GnT-IVs (MGAT4A-C)

GnT-IV catalyzes the transfer of a GlcNAc residue to α1,3-mannose via the β1,4-linkage (Figure 1) [75]. This enzyme was purified from bovine small intestine [76] and then its cDNA was subsequently cloned [77]. GnT-IV has three isoforms designated GnT-IVa–c (*MGAT4A-C*). No enzymatic activity has yet been confirmed for GnT-IVc but in GnT-IVa- and -IVb double-null mice, GnT-IV activity was completely abolished in all tissues tested [78]. We therefore now only focus on these two enzymes. GnT-IVa is distributed in limited tissues, with high expression in the pancreas and intestines, while GnT-IVb is ubiquitously expressed throughout the body. The GnT-IVa isoform displays higher affinity toward both donor and acceptor substrates than GnT-IVb [79], suggesting that GnT-IVa is the dominant GnT-IV enzyme in the tissue where it is expressed. In GnT-IVb (*Mgat4b*)-null mice, compensative upregulation of the *Mgat4a* gene was observed in various tissues [78], which explains the very mild phenotypes in GnT-IVb-null mice. Regarding its function, GnT-IVa is well known to be involved in insulin secretion and glucose metabolism in the pancreas, as described below [80].

GnT-IVs were also reported to be involved in cancer but the detailed functions in cancer cells are poorly understood. In some cancer cells, GnT-IVa or GnT-IVb is highly dysregulated including in choriocarcinoma [81], pancreatic cancer [82] and hepatocarcinoma [83] and it has generally been suggested that the product glycan of GnT-IV promotes invasion and metastasis. However, further studies are needed to clarify the functional roles of GnT-IVs and their product glycans in cancer progression.

GnT-IVa is highly expressed in the pancreas and its involvement in type 2 diabetes has been well studied. GnT-IVa-deficient mice were found to exhibit hyperglycemia, reduced insulin levels, and abnormal glucose tolerance [80]. This was found to be caused by the dysfunction of a key glucose transporter, GLUT2. *N*-glycans of GLUT2 were shown to be modified by GnT-IVa and increased galactosylation on this branch is critical for the binding to galectin and the cell surface retention of GLUT2. In GnT-IVa-null pancreatic β cells, GLUT2 was internalized and functionally impaired. Furthermore, a high-fat diet, an important causative factor of insulin resistance, downregulated GnT-IVa expression in the pancreas due to abnormal export of the key transcription factors forkhead box protein A2 (FOXA2) and hepatocyte nuclear factor 1-alpha (HNF1A) from the nuclei [84]. In addition, human type 2 diabetic patients showed the decreased expression of GnT-IVa. These findings clearly show that the GnT-IVa enzyme is critically involved in glucose metabolism and the onset of diabetes. Based on these studies, GnT-IV is considered to provide its target proteins with galectin ligands, similarly to GnT-V. Considering that both galectin [85] and GnT-V (discussed above) are involved in cancer progression, cell-proliferating and invasive effects on cancer cells are also suggested for GnT-IV, similarly to GnT-V. Future studies should reveal how GnT-IVa and –IVb regulate cancer development and metastasis.

### 2.4. Fut8 (FUT8)

Fut8 catalyzes the transfer of a fucose (Fuc) residue to the innermost GlcNAc residue via the α1,6-linkage to make the so-called “core fucose” structure (Figure 1) [86]. This structure is abundant in *N*-glycans and plays critical roles in numerous physiological and pathological processes, including cancer development and therapeutics [87]. 

This enzyme was first identified by Harry Schachter’s group in the 1980s [88]; our group subsequently purified it from a human gastric cancer cell line and porcine brain and cloned their cDNAs [89,90]. Fut8 is widely expressed in various mammalian tissues, except for liver [91] and like GnT-III, it is also aberrantly upregulated during hepatocarcinogenesis [92]. Moreover, the aberrant upregulation of *FUT8* in non-small-cell lung cancer was reported to be correlated with the poor clinical outcomes [93,94], suggesting the involvement of Fut8 in cancer development and its potential as biomarker.

As expected by the widespread expression and abundance of core fucose, Fut8 (*Fut8*)-null mice show multiple phenotypes including semilethality [95], the development of emphysema [95,96], dysfunction in the brain [97,98] and impaired immunity [99,100]. Some of these phenotypes are attributed to the dysfunction of growth factor receptors, such as epidermal growth factor (EGF) and transforming growth factor-beta (TGFβ) [6,101]. In addition, several reports have revealed that core fucose is required for growth factor-dependent cell proliferation in disease models such as lung cancer [102] and liver regeneration [103]. These findings strongly suggest that Fut8 promotes cancer growth. In fact a recent report demonstrated that Fut8-null mice exhibited reduced cancer growth in a chemical-induced hepatoma model [104]. Moreover, Fut8 or core fucose is aberrantly upregulated in several types of cancer in addition to hepatoma and lung cancer, such as breast [105] and prostate cancers [106,107]. 

Core fucosylation has already been clinically applied as a biomarker for the detection of cancer. Since Fut8 is aberrantly elevated in hepatocellular carcinoma compared with nearly no expression in normal liver, we can assume that liver-derived core fucosylated proteins in serum would be utilized for the diagnosis of liver cancer. In fact, fucosylated alpha-fetoprotein (AFP), AFP-L3, has been developed as a biomarker and approved for the early detection of liver cancer [108], being the most successful glyco-related cancer biomarker to date [2,109]. AFP itself is an oncofetal protein and was first found to be a marker of hepatoma [110]. However, the serum AFP level is also increased in other nonhepatoma diseases such as liver cirrhosis and acute and chronic hepatitis, which makes it difficult to diagnose primary hepatoma at an early stage using AFP alone. AFP glycoforms can be separated by using a fucose-specific plant lectin, *Lens culinaris* agglutinin (LCA), to give L1, L2 and L3 fractions and AFP-L3 is mostly expressed in the sera of primary hepatoma patients, but not in cases of liver cirrhosis. Based on this finding, AFP-L3 was approved by the US Food and Drug Administration (FDA) as a biomarker for the early detection of hepatocellular carcinoma. Our glycomic analysis revealed that L3 showed the highest level of core fucose among the three fractions [111]. Furthermore, fucosylated haptoglobin was also identified as a marker for various types of cancer [112,113]. Our site-specific glycomic analysis revealed that both core fucose and Lewis types of fucose that is synthesized by other Futs expressed on haptoglobin, are increased in various cancers [114]. Again, these results highlight the importance of core fucose for understanding the roles of glycans in liver cancer and their therapeutic applications.

The involvement of core fucose in antibody cancer therapy has also been reported. Antibody-dependent cellular cytotoxicity (ADCC) has a central role in cancer therapy using antibodies [115]. The removal of core fucose from IgG-Fc *N*-glycans was found to enchance ADCC activity by approximately 50-fold [116,117]. Sialylation and bisecting GlcNAcylation of IgG-Fc have also been shown to have effects on its efficacy [118] and *N*-glycan decoration is also known to be critical for the half-life in sera and the antigenicity of biopharmaceuticals [119]. These results underscore the importance of *N*-glycan branching, especially core fucosylation, for effective and safe therapy using biopharmaceuticals.

## 3. Regulation of *N*-glycan Branches in Cancer by Several Pathways

### 3.1. Genetics

Although the specific factors involved in transcriptional regulation of the genes encoding *N*-glycan branching enzymes are not well understood, some transcription factors were found to be critical for transcriptional activation of these genes in cancer cells. Oncogenic upregulation of the GnT-V gene (*MGAT5*) is the best studied among the branching enzymes described above. It was found that the Src-Raf-Ets2 pathway is involved in GnT-V activation during the transformation of BHK cells by Rous sarcoma virus [120]. Similarly, the same group reported that the Her-2/neu oncogene upregulates GnT-V in 3T3 fibroblasts via the Ras-Raf-Ets pathway [121]. Our group independently found that the Ets-1 oncogenic transcription factor directly transactivates the *MGAT5* promoter in human bile duct carcinoma cells [122] and that the Ets-1 levels were highly correlated with the levels of GnT-V expression in various cancer cell lines [123]. These reports suggest that the oncogenic Ets pathway is directly involved in GnT-V transactivation. 

Other transcription factors responsible for the regulation of *N*-glycan branching genes have also been reported, although their roles in oncogenesis are still not fully understood. For example, GnT-IVa transcription is highly activated by the liver- and pancreas-specific transcription factors HNF1A and FOXA2 [84]. In contrast to GnT-IVa, a GWAS study identified HNF1A as a negative regulator of *FUT8* transcription [124], which may explain why *FUT8* is barely expressed in the liver. The tissue-specific expression of GnT-IX is regulated by the specific transcription factors NeuroD1 and CCCTC-binding factor (CTCF) [64,65] but their contributions to the cancer-related upregulation of GnT-IX are still unclear. Intriguingly, cell density on a culture dish affects the transcription of the GnT-III gene (*MGAT3*) and high-cell-density conditions were found to induce *MGAT3* transcription. In addition, E-cadherin-α-catenin complex was shown to be involved in this process [125]. Meanwhile, Wnt/β-catenin signaling was also reported to regulate GnT-III expression [126,127]. Identification of the specific transcription factors for the oncogenic transcription of *N*-glycan branching genes is an interesting topic for future research.

### 3.2. Epigenetics

When considering the topic of genetic regulation of the *N*-glycan branching enzymes in cancer, epigenetic factors should not be overlooked. Epigenetics involves the structural adaptation of chromosomal regions beyond changes in DNA sequence and numerous cancerous changes in gene transcription and genome organization have been shown to be highly dependent on epigenetic mechanisms [128,129]. Structural adaptations of chromosomal regions are mediated by DNA modifications (mainly methylation in CpG), histone modifications, noncoding RNA including micro-RNA (miRNA) and ATP-dependent chromatin remodeling [128]. We here focus on how *N*-glycan branching genes are epigenetically regulated in cancer, particularly by DNA methylation, histone modifications and miRNAs.

DNA methylation often occurs at CpG sites and methylation of a CpG cluster at an upstream promoter region (CpG island) is well known to silence gene transcription [130]. The methylation of a CpG island allows methyl-CpG-binding proteins to bind and recruit other chromatin silencing proteins such as histone deacetylases (HDACs). DNA methylation used to be considered stable but the recent identification of ten-eleven translocation (TET) family proteins as methylated CpG conversion enzymes has required re-evaluation of this concept, leading to the current view that DNA methylation is more dynamic [131]. Some of the *N*-glycan branching genes were also found to be silenced by DNA methylation or activated by DNA hypomethylation. For example, DNA hypomethylation of the *MGAT3* promoter is involved in its upregulation in ovarian cancer cells [132]. *MGAT3* gene is also regulated by DNA methylation in other types of cancer and also during the process of EMT [31,74]; in addition, GnT-IV genes (*MGAT4A* and *4B*) were reported to be regulated by DNA methylation in pancreatic cancer [82]. Effects of altered DNA methylation on global *N*-glycan profiles including core fucose and β1,6-branch were also studied in some cancer cells [133]. These results highlight the importance of epigenetic DNA methylation for understanding cancerous changes in *N*-glycan branching.

Histone tails are usually subjected to various forms of modifications (e.g., methylation, acetylation, *O*-GlcNAcylation, and ubiquitination). These histone modifications are now considered to be pivotal for the epigenetic regulation of gene transcription [134]. The mechanisms by which the *N*-glycan branching genes are regulated by histone modifications in cancer cells are less understood than other epigenetic mechanisms [135]. GnT-IX (*MGAT5B*) is the best studied gene regarding its epigenetic mechanism [64,65]. Histones around *Mgat5b* transcription start sites are modified in a neural-cell-specific manner, which is subsequently required for the efficient binding of the specific transcription activators NeuroD1 and CTCF [64,65]. Such tissue-specific chromatin regulation of *Mgat5b* is mediated by specific chromatin modifiers such as HDAC11, *O*-GlcNAc transferase (OGT) and TET3 [64]. In future studies, it would be interesting to investigate the as-yet-unknown mechanisms involving histone modifications for the other *N*-glycan branching genes in cancer cells.

An increasing number of reports on miRNA-dependent mechanisms of glycan expression have been published. miRNAs are short noncoding RNAs that negatively regulate gene expression by mRNA degradation or translation blockade [136]. As expected by their abundance (over 1,000 miRNAs have been identified in humans), many glyco-genes are also direct and indirect targets of miRNAs. *FUT8* transcription has been reported to be regulated by several miRNAs in cancer cells. For example, miRNA-198 shows a negative correlation with poor clinical outcomes in colorectal cancer and *FUT8* was found to be a direct target of this miRNA [137]. In addition, the miRNA-198-mediated suppression of *FUT8* inhibited the proliferation and invasion of colorectal cancer cells. In hepatoma cells, *FUT8* was also found to be suppressed by miR-122 and miR-34a [138]. Recently, the GnT-IVa gene (*MGAT4A*) was shown to be silenced by miR-424 in epithelial cells, which is involved in cell cycle progression [139]. The same group has also reported several interesting findings regarding the relationships between glyco-genes and miRNAs, such as comparison of the levels of glycogenes and miRNAs in various cancer cells and prediction of miRNA binding sites in glycogene 3’-UTRs [140,141,142].

### 3.3. Nucleotide Sugar Metabolism

Nucleotide sugars (e.g., UDP-GlcNAc and GDP-Fuc), the donor substrates for glycosylation reactions, are essential nongenetic factors for glycan expression. Most *N*-glycan branching enzymes utilize UDP-GlcNAc, which is the end-product of the hexosamine pathway. Because this pathway integrates the metabolism of glucose, glutamine, acetyl-CoA and UTP [143], cellular nutrient conditions are assumed to have a major impact on it. The K_m_ values for UDP-GlcNAc are highly variable among GnTs with GnT-V possessing the highest [144], meaning that the synthesis of *N*-glycan branching can be influenced by nutrient flux. In addition, importantly, it is well known that sugar and energy metabolism is often altered in cancer cells compared with that in normal cells [145]. We previously developed methods for the simultaneous quantification of various nucleotide sugars by using ion-pair reverse-phase HPLC and found significant differences in the levels of nucleotide sugars between breast and pancreatic cancer cells [146]. Our mass isotopomer analysis further supported the notion that UDP-GlcNAc metabolism is regulated in a cell-type-specific manner [147]. These techniques are helpful to further understand how nucleotide sugar metabolism is involved in cancerous changes in *N*-glycan branching.

We also identified a unique regulatory mechanism of nucleotide sugars involving a phosphodiesterase. We biochemically purified and identified ectonucleotide pyrophosphatase/phosphodiesterase 3 (ENPP3) as an endogenous inhibitor of the GnT-IX enzyme [148]. Subsequent characterization revealed that ENPP3 hydrolyzes UDP-GlcNAc and generates UMP, a potent inhibitor of the GnT-IX reaction. Furthermore, knockdown of ENPP3 resulted in a change in the global cellular glycan profile, suggesting that ENPP3 globally regulates cellular glycosylation. Similar mechanisms probably regulate nucleotide sugars in normal and cancer cells, leading to indirect regulation of the cellular glycosylation system.

### 3.4. Subcellular Localization and the Cleavage of Branching Enzymes

The cell biological properties of glycosyltransferases such as their subcellular localization and cleavage/shedding, also critically regulates their cellular activity. The *N*-glycan branching enzymes are considered to be localized in the Golgi apparatus like many other glycosyltransferases. Although no reports have yet been published showing that their subcellular localization is altered in cancer cells, cancer-specific changes in the localization of glycosyltransferases have been shown to have a significant impact on the cellular glycosylation system [149,150]. Notably, our group reported that caveolin-1 appeared to regulate the localization and cellular activity of GnT-III in hepatoma cells [151], leading to the possibility of bisecting GlcNAc being involved in caveolin-1-related cancer phenotypes [152].

Recently, a Golgi-resident protease, SPPL3, was found to be responsible for the physiological shedding of various glycosyltransferases including GnT-V [50,51]. The overexpression of SPPL3 was found to suppress cellular glycosylation, especially the late-stage maturation of *N*-glycosylation and SPPL3-deficient cells conversely showed phenotypes of hyper-glycosylation. These SPPL3-dependent changes in glycosylation were partially attributed to the change in GnT-V activity. These results clearly showed that the cleavage and shedding of GnT-V by SPPL3 suppress GnT-V activity. Although the consequences of shedding of other glycosyltransferases by SPPL3 are still unclear, it is strongly expected that SPPL3 (down) regulates multiple enzymes involved in the cellular glycosylation system. It would also be interesting to determine the relationships between the SPPL3-mediated shedding of *N*-glycan branching enzymes and cancer biology in future studies.

**Figure 4:**
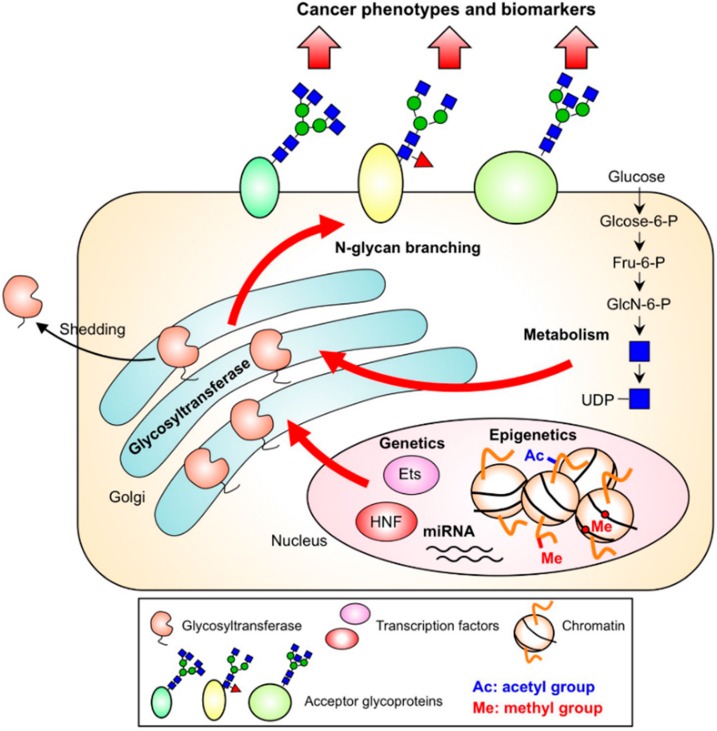
Overview of the mechanisms regulating the synthesis of *N*-glycan branches in cancer cells.

The multiple mechanisms regulating *N*-glycan branching in cancer cells described here are summarized in Figure 4 and the references for the upregulation of the *N*-glycan branching enzymes in various cancer types are listed in Table 1. Notably, other mechanisms are also involved in regulation of *N*-glycan branching such as trafficking rate of acceptor proteins, regulation of nucleotide sugar transporters, solvent accessibility at the sites to be modified [153] and crosstalk to other posttranslational modifications. In fact, multiple *N*-glycan changes were observed in colorectal cancer in a stage-specific manner and *N*-glycan branching was found to also be regulated by cellular EGFR-status [154]. Multiple factors should be considered for better understanding how *N*-glycan branching is dysregulated in cancer.

## 4. Potential Clinical Applications as Drug Targets

As described above, the changes in *N*-glycan branching are expected to be good targets for cancer therapy, including as biomarkers for early detection and anticancer drugs. To develop glyco-related biomarkers, antibodies or lectins are currently used for glycan detection. Unfortunately however, the low antigenicity of glycans and the low affinities of lectins often make it difficult to detect target glycans with high sensitivity and specificity. Technical advances will be needed to detect target glycans for clinical applications. Glycomic and glycoproteomic techniques using mass-spectrometry have been greatly improved [155,156]. Furthermore, a click chemistry approach using a sugar analog is one unique and promising strategy for this [157,158]. This approach can specifically label and tag target sugars in cells by using alkynyl- or azide-sugars as bioorthogonal chemical reporters. Although this metabolic approach is highly specific and is very promising for the selective detection of a target sugar, there are still some shortcomings of using non-natural sugars, such as cytotoxicity and low efficiency of labeling [159]. In addition, because a sugar analog is broadly incorporated into various types of glycan, we are currently unable to label a specific branch or specific part of glycans. The development of a new type of sugar analog is thus required to apply this click strategy to clinical research, such as for biomarker discovery.

Chemical inhibitors or activators of the *N*-glycan branching enzymes are considered to be useful as potential anticancer drugs, as well as tools for basic research. In particular, GnT-V inhibitors would be of great interest regarding the development of anticancer drugs. To date, no specific and potent inhibitors of these enzymes have been established, although chemically designed substrate analogs were shown to have inhibitory activities toward GnT-V or GnT-III [160,161]. The current lack of data on the tertiary structure of branching enzymes, except for Fut8 [162], makes it difficult to design *in silico* chemical modulators of their activity. An effective assay system to measure their activity for high-throughput screening also remains to be established. Solving their crystal structures and identifying their inhibitors/activators are difficult but meaningful challenges for future research.

## 5. Conclusions

In this review, we have provided an overview of how *N*-glycan branches are expressed and function in cancer cells. We know that the synthesis of *N*-glycan branches in cancer cells is regulated by multiple mechanisms encompassing genetics, epigenetics, the localization and shedding of the branching enzymes and the levels of donor substrates. Although our knowledge on this field has been increasing, there are still obstacles to its clinical application. To overcome these obstacles, technical breakthroughs for next-generation glycobiology research are needed, such as highly sensitive and protein-selective detection of target glycans and the development of novel chemical tools. A deeper understanding of the functions and properties of the branching enzymes is also required, such as their precise subcellular localization, mechanisms of protein-selective modifications and the nongenetic regulation of their expression. Future studies on these issues could eventually lead to the development of new strategies to combat cancer.

## Figures and Tables

**Table 1:** Branching enzymes and their involvement in cancer.

Enzyme	Gene	Glycan	Dysregulation in cancer	Functional output in cancer
GnT-III	*MGAT3*	Bisecting GlcNAc	Hepatoma [18], ovarian cancer [19], leukemia [20]	Suppression of metastasis [21], decrease in tumor growth [22], suppression of EMT [30,31] tumor growth [33,34]
GnT-IVa-c	*MGAT4A-C*	β1,4-branch	Choriocarcinoma [81], pancreatic cancer [82], hepatoma [83]	Invasion [81], metastasis [83]
GnT-V	*MGAT5*	β1,6-branch on α1,6-mannose	Gastric [54], esophageal [55], colon [56], liver [57] cancers	Angiogenesis [47], tumor growth [52,53]
GnT-Vb (IX)	*MGAT5b*	β1,6-branch on *O*-mannose	Neuroblastoma [72], prostate cancer [73]	Decreased adhesion, increased migration [67]
Fut8	*FUT8*	Core fucose	Hepatoma [92], lung [93,94], breast [105], prostate [106,107] cancers	Tumor growth [93,94,102,104], tumor biomarker [108], antibody therapy (ADCC) [116,117]

## Abbreviations

The following abbreviations are used in this manuscript:

Fuc	Fucose
Fut	Fucosyltransferase
Gal	Galactose
GlcNAc	*N*-Acetylglucosamine
GnT	*N*-Acetylglucosaminyltransferase
miRNA	microRNA
Sia	Sialic acid
